# Brief introduction of medical database and data mining technology in big data era

**DOI:** 10.1111/jebm.12373

**Published:** 2020-02-22

**Authors:** Jin Yang, Yuanjie Li, Qingqing Liu, Li Li, Aozi Feng, Tianyi Wang, Shuai Zheng, Anding Xu, Jun Lyu

**Affiliations:** ^1^ Department of Clinical Research The First Affiliated Hospital of Jinan University Guangzhou Guangdong China; ^2^ School of Public Health Xi'an Jiaotong University Health Science Center Xi'an Shaanxi China; ^3^ Department of Human Anatomy Histology and Embryology, School of Basic Medical Sciences, Xi'an Jiaotong University Health Science Center Xi'an Shaanxi China; ^4^ School of Public Health Shaanxi University of Chinese Medicine Xianyang Shaanxi China; ^5^ Xianyang Central Hospital Xianyang Shaanxi China; ^6^ Department of Neurology The First Affiliated Hospital of Jinan University Guangzhou Guangdong China

**Keywords:** big data, data mining, database, method, technology

## Abstract

Data mining technology can search for potentially valuable knowledge from a large amount of data, mainly divided into data preparation and data mining, and expression and analysis of results. It is a mature information processing technology and applies database technology. Database technology is a software science that researches manages, and applies databases. The data in the database are processed and analyzed by studying the underlying theory and implementation methods of the structure, storage, design, management, and application of the database. We have introduced several databases and data mining techniques to help a wide range of clinical researchers better understand and apply database technology.

## 1 INTRODUCTION

In the era of the big information explosion, the speed of information generation is increasing day by day, and the world's information is massively produced.^1^ In the past few years, to Big Data has become one of the most‐used vocabulary in the industrial sector, finance, and healthcare.^2^, ^3^ Most areas have begun to use big data to analyze and discover new value.^4^, ^5^ The information brought by big data is also changing the ecosystem of medical education and medicine. The amount of data collected and stored digitally is growing exponentially.^1^, ^6^ The medical industry is producing a large number of data every day, which is an important area of big data's application. In order to provide patients with the best services and care, medical institutions in many countries have proposed various modes of medical information systems.^7^ Therefore, how to better develop and utilize the huge medical big data has become the focus of attention, and promoting the research and application of medical big data has become a key factor in modern medical research.

Big data is an abstract concept. It is usually explained that big data refers to the data integration which is difficult to deal with the existing database management tools, which has both massive characteristics and complexity characteristics. Big data are frequently characterized as the five Vs—volume, velocity, variety, value, and veracity.^8^, ^9^, ^10^ Volume is “huge in volume” with the massive generation and collection of data, the scale of the data has become larger and larger, and has gone beyond traditional storage and analysis techniques; velocity is “speed,” that is, big data's timeliness, which means that data collection and analysis must be carried out quickly and on time; variability is “a wide range of data types,” including semistructured and unstructured data, such as audio, video, web pages, and text, as well as traditional structured data; Value is “value,” which is mainly reflected in the low density of value and the commercial value is high. Veracity which emphasizes that meaningful data must be true and accurate. The key question when using big data is how to find value from a large, rapidly generating, and diverse data set.^11^, ^12^ The computational analysis of integrating databases has become the basic method of medicine and molecular biology.^13^

Medical data have the characteristics of disease diversity, heterogeneity of treatment and outcome, and the complexity of collecting, processing, and interpreting data.^14^ With the development of medical information, a large number of digital data has been produced in the process of medical service, health care, and health management, forming medical big data.^15^ Medical big data come from a variety of sources, such as administrative claims records, clinical registration, electronic health records, biometric data, patient report data, and more.^16^, ^17^ There are many values in big data applications and data collection in healthcare systems. For example, people with diabetes use mobile devices to communicate with each other, share information or search for information, thus, forming a large group of big data networks.^18^ The US Department of Health and Human Services has issued a policy to increase the transparency of the US healthcare system, which constitutes big data sharing for many patients, physicians, and medical‐related information.^19^ Faced with a huge amount of different types of electronic data, new requirements for R&D‐related electronic products have been put forward to adapt to complex and competitive big data and its logical way.^20^, ^21^ From the massive electronic medical record data, we found that the new efficacy of existing drugs—metformin for cancer treatment can also be used to treat diabetes.^22^

Medical big data have several unique characteristics that differ from big data in other disciplines: medical big data are often difficult to obtain^10^; are usually based on protocols, collected and relatively structured^23^; and when analyzing data and interpreting results, the role of professional knowledge may be dominant^24^; time‐dependent mixing.^3^ Medical data are large in scale, extremely fast in update, polymorphic, incomplete, and time sensitive.^25^ The construction of a big data platform will facilitate the remote consultation, easy operation, low cost, increase global cooperation to promote clinical practice, education and scientific research, help the global precision medicine transformation application and the emergence of new health management model.^26^, ^27^

## 2 MEDICAL PUBLIC DATABASE OVERVIEW

Today's society produces massive amounts of data all the time. Database technology is a software science that researches, manages, and applies databases. The data in the database are processed and analyzed by studying the basic theory and implementation methods of the structure, storage, design, management and application of the database. The main medical public databases are described in Table 1.

**TABLE 1:** Medical public database overview

Databases	Range	Patients	Cost
SEER	Tumor	USA	Partially free
MIMIC	Intensive care unit	USA	Free
CHNS	Health and nutrition	China	Partially free
HRS	Ageing health	Global	Free
Dryad	Medicine, biology, ecology	Global	Free
UK Biobank	Biomedical	UK	Free
BioLINCC	Blood and cardiovascular	USA	Free
GEPIA	Cancer genomics	USA	Free
TCGA	Cancer genomics	USA	Free
TATGET	Childhood cancer	USA	Free
eICU‐CRD	Intensive care unit	USA	Free
GEO	Genomics data	USA	Free
GBD	Burden of disease	Global	Free

BioLINCC, biologic specimen and data repositories information coordinating center; CHNS, China health and nutrition survey; eICU‐CRD, eICU collaborative research database; GBD, global burden of disease; GEO, gene expression omnibus; GEPIA, gene expression profiling interactive analysis; HRS, health and retirement research; MIMIC, medical information mart for intensive care; SEER, Surveillance, Epidemiology, and End Results; TATGET, therapeutically applicable research to generate effective treatments; TCGA, the cancer genome atlas.

### 2.1 Surveillance, epidemiology, and end results (SEER)

To reduce the cancer burden of the population, the National Cancer Institutes established a monitoring, epidemiological, and final results database (SEER) for cancer patients in 1973.^28^ This is one of the most representative large tumor databases in North America, which covers approximately 28% of the US population.^29^, ^30^ SEER has collected information on the incidence, prevalence, mortality, and other evidence‐based medicines of cancer patients in some US states and counties for decades, providing valuable information on cancer diseases for the majority of clinical medical staff.^31^ Especially, it provides a broad path for the study of malignant tumors and rare tumors. At the beginning of the establishment of SEER, there were only a few registration stations in several regions. The number of registration stations has now expanded to 18.^32^ These registration stations operate using SEER*STAT software and are submitted to NCI for biennial frequency statistics and aggregation, and then publicize the cancer‐related information of the covered population to the United States and the world.

The SEER database has a large sample size, high quality, and strong statistical power, which can provide tumor‐related researchers with high clinical reference value data. Researchers can obtain partial data through the application of the account number. There are three ways to obtain data from the SEER database: the first way is obtained by SEER*Stat software, this method is the simplest and widely used; the second method is download the compressed file from the SEER official website, extract the binary data after decompression, and then use software such as R is converted to data in the normal format and this method requires the user to have certain software knowledge; the last way is used by applying to the management personnel for DVD discs and using SEER*Stat without high‐speed internet support. Radiation therapy and chemotherapy variables in public databases have been removed since the November 2016 data submission. These variables can be obtained after signing an additional data usage agreement. The protocol describes the integrity of the chemoradiotherapy treatment variables and the potential bias in the use of chemoradiotherapy data.

The SEER database is one of the most representative tumor databases in North America, and some of the data are free to the public.^33^ Although the SEER database has some shortcomings, such as family history of cancer patients, genetic history, genes, disease recurrence, and adjuvant chemotherapy, are not included, the SEER database is still a good source of data, providing high quality for clinical researchers.^34^, ^35^, ^36^ Clinical information helps clinical researchers provide efficient, convenient, and clear access to data.

### 2.2 Medical information mart for intensive care (MIMIC)

Severe medicine is a discipline that studies the characteristics and regularity of any injury or disease that leads to the development of the body in the direction of death, and treats severe diseases. The focus of this discipline is on the monitoring of critically ill patients, the implementation of organs for organ dysfunction or debilitating organs. Support, so that patients can win the time to remove the cause under the condition of ensuring oxygen delivery and maintaining organ function. As we all know, intensive care unit (ICU) is in a very special important position in the hospital, and undertakes the treatment of patients with serious diseases.^37^, ^38^ The level of diagnosis and treatment is also one of the important indicators for modern measurement of hospital level. The era of big data provides an unprecedented opportunity for the study of critically ill patients. By strengthening basic and clinical research, making full use of big data and artificial intelligence is the development trend of future critical medicine.

In order to promote the work of intensive medical research, the MIMIC (Medical Information Mart for Intensive Care) database jointly issued by the Massachusetts Institute of Technology's Computational Physiology Laboratory, Beth Israel Dikang Medical Center, and Philips Medical supported by the National Institutes of Health. The clinical diagnosis and treatment information of more than 40 000 real patients living in the ICU of the Beacon Israel Dikang Medical Center from 2001 to 2012 was collected.^39^ The database has a large sample size, comprehensive information, long patient tracking, and can be used free of charge, providing a wealth of resources for the study of critical care.^40^, ^41^ It provides abundant resources for the study of severe medicine, and solves the problem that clinical medical workers suffer from a large number of systematic clinical diagnosis and treatment data for scientific research status quo. The MIMIC database is constantly updated and the latest release is MIMIC‐III version 1.4 (release notes available from https://mimic.physionet.org/about/releasenotes/).^42^, ^43^ Patients information for the database come from two different intensive care information systems: the Philips Care Vue Clinical Information System (https://mimic.physionet.org/mimicdata/carevue/) and the IMD Soft Meta Vision ICU System (https://mimic.physionet.org/mimicdata/metavision/). From 2001 to 2008, the Philips Care Vue clinical information system was used to track patients for a minimum of 4 years; from 2008 to 2012, the IMD Soft Meta Vision ICU system was used to track patients for a minimum of 90 days.

The MIMIC database involves coding work during its use, which is a challenge for clinicians. On the GitHub platform (https://github.com/MIT-lcp/mimiccode), there is an open‐source code package for analyzing patient characteristics that can be downloaded and used free of charge by researchers around the world. When bugs or improvements are found, you can modify it yourself, and then you can pull request, when the platform merge, you can successfully share your modified code package to the world, other users can also use it for free. The MIMIC database has great support for the research in the fields of critical medicine, evidence‐based medicine, clinical big data mining, and medical monitoring equipment data analysis, and has achieved fruitful results.

The MIMIC database is open to the world and collects the actual medical treatment of more than 40 000 patients in the ICU of the Beth Israel Dikang Medical Center for 12 years. The sample size is large and the information is comprehensive. Github provides open‐source code for researchers all over the world to use. In short, the MIMIC database provides excellent support for all aspects of clinical research.

### 2.3 China health and nutrition survey (CHNS)

The CHNS project, the China Resident Health and Nutrition Survey, is an open public platform (http://www.cpc.unc.edu/projects/china). The project is a cohort of international collaborations conducted by the University of North Carolina at Chapel Hill Population Center in conjunction with the Center for Nutrition and Health of the Chinese Center for Disease Control and Prevention.^44^ The study aims to explore how China's socioeconomic transformation and family planning policies have affected the health and nutritional status of the country over the past 30 years. The research includes the status and changes of community organizations, family and individual economic, demographic and social factors. The research team for this survey is an international research team composed of researchers in the fields of nutrition, public health, economics, sociology, and demography. The project began in 1989 and carried out project research and data compilation and release in 1989, 1991, 1993, 1997, 2000, 2004, 2006, 2009, 2011, and 2015. The CHNS website updated the dataset content on 12 June 2018. The updated dataset covers vertically integrated data from 10 survey data from 1989 to 2015.^45^ The China Health and Nutrition Survey (CHNS) has shown the shift in the form of either nutrients or food items or dietary patterns and this dietary shift is associated with education, income, urbanicity, and macro food environment and policy.^46^, ^47^, ^48^, ^49^

The survey used multistage stratified cluster random sampling to collect data from 15 provinces, autonomous regions, and municipalities in China's eastern, central, and western regions.^50^, ^51^ As of August 2018, a total of 220 community samples, 7200 family samples and 30 000 resident samples were included. The survey data include community surveys, household surveys, and personal survey data. Personal and household survey data include basic demographics, health status, nutritional and dietary status and health indicators, and medical insurance.^52^

The family data and personal data in the CHNS data are freely available to the public on the official website of CHNS. The community data can be obtained through the community‐level data use agreement and completed online (Data Linkage Request Form). Paid access, the fee standard is 330 dollars. Researchers should really apply the CHNS database information, and a detailed reading to fully understand the CHNS project research documents is a necessary prerequisite. The CHNS database website provides a clear and detailed study description document. The questionnaires, database descriptions, ID variable names, etc. of the calendar year are there in the CHNS database description file.

CHNS, a longitudinal cohort study of international cooperation, began in 1989 and has conducted 10 surveys until the year of 2015. The research covers data on the health and nutritional status of Chinese residents at the individual level, family level, and community level. Since then, Chinese national health, nutrition, medical, economic, social and other research has provided more comprehensive data support.^53^ The official website of CHNS covers the details of the research, and the website not only updates the number of studies dynamically. According to the research, links related to research are provided, and the existing research results based on this research can be conveniently retrieved. The CHNS research data set can be downloaded and obtained on the official website of CHNS, which is efficient and convenient.

### 2.4 Health and retirement research (HRS)

As an important measure of the level of international economic and social development, population ageing not only means an increase in the number of elderly people but also poses severe challenges to the economy and society.^54^ This has become a major social problem that cannot be ignored. There are many types of research on the health of an ageing population, data types are constantly enriched, and data reserves are growing rapidly. It is difficult to conduct an effective and comprehensive statistical analysis through traditional data collection methods.

The Health and Retirement Research (HRS), supported by the National Institute for Ageing (NIAU01AG009740) and social security management, is a longitudinal research group survey conducted by the University of Michigan since 1992 and has established a representative large sample database.^55^ More and more multidisciplinary data are provided through unique and in‐depth interviews every 2 years for participants over 50 years of age. The HRS database provides valuable information for researchers, such as society and healthcare, to use it to address important issues related to ageing challenges and opportunities.^56^, ^57^, ^58^ International substudies that have evaluated global ageing and similarities have also emerged, including research in Mexico, the United Kingdom, Europe, South Korea, Japan, Ireland, China, Indonesia, Costa Rica, and New Zealand, while developing to India, Brazil, Africa, Scotland, and Canada to form a global ageing health big data.

The HRS database has a large sample size, high quality, and is complex. In order to make the data easier to study, the HRS data are classified into public data and sensitive/restricted data. Anyone can create an account on the HRS data download site to get public data while restricting data and sensitive health data requires a separate application. The HRS database can be accessed in seven areas, including biennial data products, vertical data, nonelected year studies, sensitive health data (requires additional registration), researcher contributions, RAND's contribution data, and cognitive, economic projects. Each subdataset file can be read by three different statements: SAS, SPSS, or Stata.

The HRS database is a database of resources related to ageing in the United States regarding changes in health and economic environment. Most of the public data in this database are freely available through user registration. Its multidisciplinary data focuses on surveys of income and wealth, health, awareness, and use of health services, work and retirement, and contact with the family. Since 2006, data collection has expanded to include biomarkers and genetics as well as greater depth of psychology and social background. This mixed‐economy, health and psychological information database provides the unprecedented potential for researchers' work.^59^, ^60^ The HRS database can help researchers in all disciplines to obtain more convenient, efficient, and clear data to improve work efficiency.

### 2.5 Dryad

With the advent of the era of big data, data reusability and data sharing policies are attracting global attention. The infrastructure and related regulations for data management and data sharing have been rapidly developing in the past decade. Since 2003, the National Institutes of Health has required all large funds to fund research projects to disclose their data. PLOS One, the world's largest open‐access journal, requires authors to submit their data to a public database platform while the article is published. The BMJ Publishing Group recommends that authors store data in the Dryad database while submitting the manuscript.^61^, ^62^, ^63^ As a large and robust data sharing platform, Dryad is a model for realizing data circulation and improving data reuse.

The Dryad database was funded by the National Science Foundation and was established in September 2008 as a nonprofit membership organization. The Dryad database stores research data in the fields of medicine, biology, and ecology. It is open to the world and can be downloaded free of charge and reused. Dryad was born out of the initiative of leading journals and scientific groups in the fields of biology and ecology, and they encourage researchers who submit journals to submit data to a professional database to store data and share data (http://dryad2.lib.ncsu.edu/pages/organization). The Dryad database helps researchers realize that data can be archived for long periods of time and open for free reuse. As of February 2018, there were more than 600 journals working with the Dryad database, with more than 60 000 data files and 2.3 million downloads (http://dryad2.lib.ncsu.edu/).

More and more journals encourage researchers to publish research data. On the one hand, encouraging the reuse of scientific research data to generate more new scientific discoveries, on the other hand, promoting the transparency and openness of medical research. Researchers publish data on Dryad for sharing. When someone searches for data through Dryad, they will find that articles published using the data help to increase the reputation and academic influence of the researcher and the publisher. At the same time, Dryad assigns each packet a globally identifiable, permanent digital object identifier (DOI) that can be used for data references. Dryad will perform a necessary check on each submitted file. For example, whether a file can be opened, whether there is a virus, whether there is a copyright restriction, and whether sensitive data are displayed. Dryad also checks the integrity and correctness of the metadata. For example, information about related publications delayed dates for data publication, index keywords, etc. Once the article is published online, the data package will be published publicly, unless the data provider chooses to post the data. Because the title, abstract, author, etc. of the article often changes during the publishing process, Dryad will confirm and update this information based on accepted or published articles.

Compared to other public database platforms, the Dryad database is more efficient in data sharing by working with many mainstream journals. By assigning DOIs to metadata, data can be referenced, increasing the scientific data utilization rate while increasing the academic reputation of researchers and publishers. Dryad has a detailed management policy for data maintenance and data disaster recovery, so data can be stored for a long time. The use of data “zero thresholds” and friendly interface also make the Dryad database more and more popular among researchers.

### 2.6 UK biobank

UK Biobank (http://www.ukbiobank.ac.uk), is the world's largest database of biomedical samples and officially opened all data to global researchers on 30 April 2017. Between 2006 and 2010, UK Biobank recruited 500 000 volunteers aged 40‐69 from across the United Kingdom to obtain baseline data, including family history, drug history, and health status.^64^ UK Biobank collected approximately 15 million biological samples of blood, urine, and saliva and performed genotyping and blood biochemical analysis on all participants.^65^, ^66^ Moreover, the database will keep track of their health and medical profile information for a long time. At the same time, the database collects all research results and provides them to other researchers. It aims to study the relationship between genetic factors, environmental factors, living habits, and other major human diseases.^66^

UK Biobank started a new medical imaging data collection program in 2014, using magnetic resonance imaging (MRI) and X‐ray technology to brain, heart, and bone of more than 100 000 volunteers.^67^ The imaging analysis is performed to establish a database of scanned images of internal organs. This will also be the most significant health imaging research in the world to date. These vast amounts of data will help researchers analyze population differences and their causes, such as cancer, heart disease, diabetes, arthritis, Alzheimer's disease, and even change scientists' perceptions of such chronic and epidemic diseases.

UK Biobank's application process has high requirements for researcher's and research institutions' research background, research purposes and research motivation, including the need to provide evidence of recently published academic results to ensure that research is conducted in good faith.

The most significant advantage of UK Biobank is that all volunteers recruited are registered with the UK National Health Service (NHS) and agree to link their medical records. This allows UK Biobank to track the health and health of all volunteers in detail through national medical data.^68^ Prospective cohort studies are important for the identification of disease risk factors and the prevention, treatment and treatment of diseases. However, too small a cohort is detrimental to the study of rare diseases and the complicated relationship between different risk factors and diseases. UK Biobank's forward‐looking and large sample size and continued integration with health records provide researchers with an excellent platform to address a variety of research issues.

The disadvantage of UK Biobank is that the sample provider must fill out a detailed basic situation questionnaire, including its name, gender, NHS number, disease information, etc., and there are inevitable privacy leaks.^69^ At the same time, the registration and application process is complicated and cumbersome, and the period is long. It may be difficult for the first‐time applicant.

We believe that UK Biobank will provide more comprehensive research data and biological sample coverage in the future, providing global researchers with more efficient and convenient resource registration, application and use services, as well as more secure information security.

### 2.7 Biologic specimen and data repositories information coordinating center (BioLINCC)

BioLINCC was established in 2008 by the National Heart, Lung, and Blood Institute (NHLBI). The Institute provides global leadership in the prevention and treatment of heart, lung, and blood diseases and supports basic, transformational, and clinical research in these areas. By establishing BioLINCC, NHLBI provides medical researchers with access to scientific data and access to biological samples, maximizing the utilization of research resources for NHLBI development and maintenance. These resources are the NHLBI biological sample library managed by the Blood Disease Resources Department since 1975 and the NHLBI database managed by the Cardiovascular Science Research Center since 2000.^70^, ^71^, ^72^

The BioLINCC public website (https://biolincc.nhlbi.nih.gov/) was established in October 2009. The site provides clinical and epidemiological research data and biological samples from more than 110 research institutes collected by NHLBI. BioLINCC is actively engaged in data sharing and is loved by many medical science and technology workers. Each year, more than 100 research project leaders apply to BioLINCC for their clinical data. A study from the affiliated hospital of Yale University School of Medicine in 2015 showed that more than 90% of users are satisfied with the data shared by BioLINCC and are suitable for conducting their clinical research using this data. Half of the users have used data to complete their research, and 67% of them have published articles with more than 1000 articles.^73^

Data and biological samples stored in the BioLINCC database are provided free of charge, but the cost of shipping of the biological samples is at the expense of the investigator. Researchers are required to submit an application to BioLINCC for review and access to the data or biological samples they are applying for. After the researcher applies to data or biological samples, the NHLBI staff will review the application materials. For the application of data resources, NHLBI mainly reviews whether the application data matches the research plan, and the ethics committee's explanation of the research plan, the ethical review is passed or exempted. BioLINCC will send an email reminder to submit the study on 1st March every year. The progress report, the researcher, can also submit a progress report on his application page at any time after the application is successful. The published article will be displayed on the research project page, where the resource is located.

BioLINCC is a collector of high‐quality medical research data and biological samples. It is the disseminator of advanced medical research concepts and research methods and is a practitioner who actively promotes global medical data sharing. Through the use of BioLINCC research resources, more and more research results are continually being produced. The disadvantage of BioLINCC is that each resource shared by BioLINCC needs to be applied separately. For applicants who want to apply for multiple research resources, the application process is complicated; when searching for biological samples, BioLINCC needs to provide the name of the biological sample for research purposes. The search method is not efficient enough for unidentified researchers. In the future, BioLINCC will also expand the field of data sharing, provide a more convenient resource application process, collect and maintain data and specimens in a “high‐efficiency‐low cost” way, and maximize the utilization of existing resources.

### 2.8 Gene expression profiling interactive analysis (GEPIA)

The use of big data analysis has facilitated the development of cancer genomics research. In essence, the cause of cancer is a genetic disease caused by differential gene expression within the cell. With the establishment and opening of many public databases, more and more researchers can access sequencing data. GEPIA (Gene Expression Profiling Interactive Analysis), a dynamic analysis of gene expression profiling data, is a newly developed web server for cancer and normal gene expression profiling and interactive analysis, filling the gaps in cancer genomics big data information and helping clinical research people use public data resources more efficiently.

GEPIA (http://gepia.cancer-pku.cn/index.html) was developed by Professor Zhang Zemin from Peking University. The RNA‐Seq data set used by GEPIA is based on the UCSC Xena project (http://xena.ucsc.edu). The project was calculated by standard pipelines and analyzed RNA sequencing expression data from 9736 tumors and 8587 normal samples from the TCGA and GTEx projects. TCGA produced 9736 tumor samples in 33 cancer types, and this project only provided 726 standard samples. The imbalance between tumors and standard data can lead to inefficiencies in various identification analyses, so GEPIA also integrates data from GTEx. The GTEx project produced RNA sequencing data for 8 000 standard samples. At the same time, the UCSC Xena project recalculated the TCGA and GTEx raw RNA‐Seq data using standard pipelines, which made the two datasets compatible. Therefore, TCGA and GTEx data can be integrated for very comprehensive expression analysis. The expression data of TCGA and GTEx are recalculated under the same pipeline and can be directly compared. GEPIA uses MySQL to create databases. The topic analysis process is done by R and PerL. Web‐based interactive display with php provides key interactive analysis of GPIIA, including tumor/normal differential expression profiling, section mapping, based on tumor type or pathological staging. Analysis modules such as analysis, patient survival analysis, similar gene detection, correlation analysis, and dimensionality reduction analysis, as well as rapid customization are used.

GEPIA is a public database developed by the Chinese. Using the GEPIA database, laboratory biologists can easily explore TCGA and GTEx data sets, find answers to questions, and test their hypotheses. In the differential analysis and expression profiling, users can easily discover genes that are differentially expressed. With the application of genetic testing, the model of tumor prognosis assessment and treatment options for immunohistochemistry‐based tumors has been gradually changed, and the more accurate classification of tumors has more important guiding significance for prognosis evaluation and treatment.

### 2.9 The cancer genome atlas (TCGA)

For a long time, tumor prevention, early screening, individualized treatment, and prognosis evaluation have always been the key issues that the medical community is committed to. As a result, the magnitude of cancer is increasing substantially, with over 20 million new cancer cases projected for 2025 compared to an estimated 14.1 million new cases in 2012.^74^ The study found that genetic variation is an important microscopic molecular cause of all tumor cells. Therefore, more and more oncology researchers began to conduct related research from the perspective of molecular genetics. By measuring the biological identity of specific gene expression, it is possible to predict tumor growth, spread, and patient survival, and to develop a targeted diagnosis and treatment plan based on gene expression.^75^ Whole‐genome sequencing and the development of bioinformatics provide new clues for cancer genome research.^76^

TCGA is a publicly funded project led by NCI in 2006. It has published phased results since 2008.^77^ In 2009, it continued to invest US$275 million, increasing various types of cancer data. By 2014, the analysis extended to 33 other types. Cancer data (including 10 rare tumors), from more than 11 000 tumor samples, data volume up to 255T, including clinical data, DNA, RNA, protein, and other multilevel data. In terms of data generation, the project achieved undisputed success. The goal of TCGA is to integrate multidimensional omics data through large‐scale, high‐throughput genome sequencing, and gene chip technology to study, define, discover, and analyze all human tumor genome changes, and finally draw a genome‐wide, multidimensional cancer genome map.^78^ TCGA provides a large amount of genomic data and related clinical data for oncology researchers, providing a large data base for finding small mutations in cancer‐related genes and studying tumor biological mechanisms, thereby, improving people's scientific understanding of cancer at the molecular level and the ability to prevent, diagnose, and treat. For example, some researchers use gene expression data and patient survival data to explore the association between the two, and then predict the patient's survival.^79^, ^80^, ^81^ TCGA includes genomes, proteomes, transcriptomes, epigenetic groups, and clinical data.^82^ These data are supported and maintained by multiple organizational structures and units.

TCGA has opened up an era of tumor molecular biology and precision medicine, providing researchers with new opportunities to study the development of cancer, allowing us to look at cancer with an unprecedented microscopic perspective, so that we can get closer to its overall picture step by step. Currently, TCGA data have been used to discover new mutations, identify intrinsic tumor types, and determine pan‐cancerous similarities and differences. At the same time, evidence of tumor evolution was collected. More and more bioinformatics tools for the TCGA database have been developed.

### 2.10 Therapeutically applicable research to generate effective treatments (TARGET)

In recent years, with the continuous development of medical level, the overall prognosis of childhood cancer has been greatly improved, but childhood malignant tumors are still the main cause of childhood death. The TARGET (Therapeutically Applicable Research to Generate Effective Treatments) database is a multiomics approach to determining molecular changes that drive the development and progression of childhood cancer. The TARGET database targets childhood tumors, and major disease items include acute lymphoblastic leukemia (ALL), acute myeloid leukemia (AML), kidney tumors (KT), model systems (MDLS), neuroblastoma (NBL), and osteosarcoma (OS). TARGET detects the genome, transcriptome, and epigenetics of specific childhood cancers through sequencing and chip technology. A multiomics approach is used to generate a comprehensive molecular alteration map for each type of cancer (change refers to changes in DNA or RNA, such as rearrangement of chromosome structure or changes in gene expression). By calculating and validating biological functions to determine which changes disrupt the functional pathways of genes, promote cancer growth, progression, and survival, thereby, identifying candidate therapeutic targets and prognostic markers from cancer‐related changes. The TARGET program originated from two pilot projects: ALL and NBL.

To date, TARGET consists of five projects, ALL, AML, KT, NBL, and OS. It is managed by NCI's Office of Cancer Genomics and Cancer Therapy Evaluation Program. ALL is one of the major types of childhood leukemia. The ALL project clarifies the comprehensive molecular characteristics to identify genetic changes in the initiation and progression of childhood cancer that are difficult to treat. AML is a cancer derived from immature white blood cells in the bone marrow or myeloblasts. About 25% of children with leukemia have AML. Through comprehensive genome‐wide identification, researchers can develop more targeted treatments based on genetic and epigenetic changes found to improve the prognosis of children with AML. KT is a kidney tumor. Children's KTs account for about 7% of childhood cancer. The vast majority are nephroblastoma. Through genome‐wide research, it is hoped that key molecules of these tumors will be discovered so that better treatments can be developed to improve the prognosis of patients. NBL is a neuroblastoma. Neuroblastoma (NB) tumors are the most common extracranial solid tumors in children, and have become one of the most important diseases that affect children's physical and mental health. The NBL project clarifies comprehensive molecular characteristics to identify genetic changes in the initiation and progression of cancer in high‐risk or difficult‐to‐treat children. OS is osteosarcoma. TARGET generates genomic data for selected pediatric cancers and provides access to discover therapeutic targets for childhood cancer and translate these findings into clinical applications.

The TARGET large database targets children's tumors, although it contains fewer types of diseases, but it is more targeted. To a certain extent, the database can help researchers conduct more in‐depth disease research and lead to more precise treatment options.

### 2.11 eICU collaborative research database (eICU‐CRD)

Severe medicine is an inevitable trend and a prominent symbol of the development and progress of modern medicine. It is an era product of the development of medical science to a fairly high level. There are many difficult problems involved in critical medicine, including the application and management of noninvasive ventilation, the rational use of antibiotics, the implementation of nutritional assessment and nutritional support, the indications for analgesia and sedatives, and the scope of application of the ICU risk assessment model.^83^ Philips Healthcare is a leading provider of ICU equipment and services, offering a teleICU service called the eICU program. After implementing the eICU plan, a large amount of data is collected and streamed for real‐time monitoring by the remote ICU team. These data were archived by Philips and converted to a research database by the eICU Institute.^84^

The eICU Collaborative Research Database (eICU‐CRD) is a large public database created by the Philips Group in collaboration with the Massachusetts Institute of Technology (MIT) Laboratory for Computational Physiology (LCP).^85^ The release of eICU‐CRD is based on the successful establishment of MIMIC‐III and expands the scope of research by providing data from multiple centers. The database consists of data from a number of ICUs in the United States. The current version is Version 2.0 and was released on 17 May 2018. The database covers routine data from more than 200 000 ICU patients in 2014 and 2015, collecting a wealth of high‐quality clinical information including vital signs, care plan documentation, disease severity, diagnostic information, and treatment information. The free availability of data will support many applications including machine learning algorithms, decision support tools, and the development of clinical research.

To obtain access to the eICU Collaborative Research Database, you must first apply for registration.^86^ The agreement stipulates that applicants do not share data with others, do not attempt to reidentify any patient or institution, and abide by the principles of collaborative research.^87^ There is a repository on GitHub to store eICU collaborative research database code, and the code for generating tables and descriptive statistics is available online (https://github.com/mit-lcp/eicu-code).

With the advent of health information networks, humans need to develop cost‐effective systems to reduce the time and effort spent recording health care data. Patients in the ICU are closely monitored throughout the hospital stay to detect changes in the condition. The patient's condition changes require the medical worker to modify the treatment plan in time. The eICU collaborative research database solves the problem that it is difficult for medical workers to have a lot of time and energy to collect a large amount of complete information, and it is free to open to medical workers all over the world.

### 2.12 Gene expression omnibus (GEO)

The GEO database is an international public function gene expression repository created by NCBI. The data have powerful inclusion and storage capabilities that allow users or researchers to submit, save, and retrieve many different types of data. GEO provides a simple submission process and format whose data source relies on data submission from researchers. GEO data submission follows the MIAME principles. The GEO database architecture not only provides researchers with a wealth of disease‐related gene expression profiles, but also provides tools for querying and downloading experiments and gene expression data, allowing users to query and download interesting research and gene expression profiles. The GEO database contains the raw data and the data set or map generated from the original data. GEO's raw data are placed in three different entity databases: platform, sample, and series.

The search results of the GEO dataset include name, description, species, platform, submitter contact, series, publication time, numeric type, and the number of samples. The search results of the GEO expression map show the expression level of a gene for all samples in the form of pictures. The experimental conditions in the search results facilitate us to observe the difference in expression levels of a gene under different conditions. Each dataset outlines its research data report and purpose, showing the number of platforms, samples, and series associated with it, from which researchers can select the research content of interest to download the data.

GEO also offers GEO2R online analysis tools. GEO2R is an interactive web tool that uses GEO2R to screen differentially expressed genes, allowing users to compare two or more groups of GEO series to identify genes that are differentially expressed under different experimental conditions, and the results are shown to be significant (sorted gene tables). GEO2R uses the GEOquery and limmaR packages from the Bioconductor project to perform a comparison of the original processed data tables provided by the submitters. Unlike GEO's other dataset analysis tools, GEO2R does not rely on collated datasets, but instead queries the original series of matrix data files.

Developed and maintained by NCBI, GEO is one of the well‐known comprehensive databases for storing and querying chip data. There are various chip technology platforms. The Gene Expression Omnibus (GEO) was created in 2000 and the last modified date is 26 July 2016. The researchers explored the potential biological value through the deep mining and analysis of the gene expression data information provided by the gene chip, and applied it to the research of gene analysis, gene expression and regulation, disease diagnosis, and drug screening. The mining and analysis of gene expression profile data help to understand the function of genes and the interactions between genes, and to analyze the genetic characteristics and functions of genes. GEO adapts to the development trend of chip database, reduces the cost of chip detection, shortens the data reading time, efficiently and rationally utilizes resources, and integrates data of more researchers.

### 2.13 Global burden of disease (GBD)

People have always been concerned about the dangers of diseases that endanger human health. Accurately grasping the burden of various diseases around the world is of great significance for understanding the degree of damage and development of diseases, improving the efficiency of health services, and promoting the health and social and economic development of residents.^88^, ^89^ In 1988, with the support of the World Health Organization (WHO) and the World Bank, funded by the Bill and Melinda Gates Foundation of the United States, the Harvard School of Public Health began research on the GBD.^90^ Subsequently, the Institute of Health Measurement and Evaluation of the University of Washington established the GBD Research Group to study the GBD.^91^, ^92^

The GBD is a comprehensive health loss study. The GBD database contains all GBD disease, risk, etiology, injury, natural injury, and sequela syndrome. Indicators that measure the GBD include: deaths, loss of life (YLLs), lifespan disability (YLDs), life‐limiting disability (DALYs), prevalence, morbidity, life expectancy, probability of death, and healthy life expectancy (HALE), maternal mortality (MMR), and total exposure (SEV). The extracted data indicators (units) include: quantity, ratio, percentage, year, and probability of death. The year of extractable data is: the annual results of all measures from 1990 to 2017, and all GBD age groups; Gender: male, female, or a combination of both. The research areas are divided into: GBD super regions, regions, countries, and selected subnational units, World Health Organization regions, World Bank income levels, etc. The data can be downloaded free of charge to help a wide range of clinical researchers.

Although the GBD database can query and download data, including many search parameters can cause problems: Query sometimes causes the file to ignore certain results specified in the query: specific age groups, years, etc.; query all locations at the same time and many or all of the reasons, age groups, years, etc. will appear incomplete data. This tool is not available for Internet Explorer 10 and earlier.

## 3 CLINICAL DATA MINING METHODS

With the advent of the information age, data mining is increasingly being used in clinical practice. With information technology, medical records and follow‐up data can be stored and extracted more efficiently. At the same time, look for potential relationships or laws from medical data to gain effective knowledge of diagnosis and treatment of patients; increase the accuracy of disease prediction, detect disease at an early stage, and improve cure rate. Different from the traditional research methods, data mining is to mine information and discover knowledge without explicit assumptions, that is, without prior research and design, the information obtained should have three characteristics: previously unknown, effective, and practical. The emergence of data mining technology is not to replace the traditional statistical analysis technology, but the extension and extension of statistical analysis methodology. Data mining methods can be divided into two categories: descriptive and predictive. Descriptive patterns characterize the general nature of data including association analysis and clusters analysis. Predictive patterns are summarized on current data including classification and regression.

### 3.1 Description

#### 3.1.1 Association analysis

Association analysis, also known as association mining, is the search for frequent patterns, associations, correlations, or causal structures that exist between project collections or collections of objects in transaction data, relational data, or other information carriers. In other words, correlation analysis is the discovery of the connection between data from large amounts of data. Shopping basket analysis is a classic example of correlation analysis. It mainly analyzes the customer's buying habits by discovering different products in the customer's shopping basket. Knowing which items are often purchased by customers at the same time can help retailers develop marketing plans. This phenomenon is the correlation between goods in the store. The association analysis consists of two steps: first, list all the high‐frequency items in the set; then, generate frequent association rules based on the high‐frequency items. The second step is the generation of association rules. According to the high frequency item group obtained in the first step, if the rule satisfies the minimum confidence, the rule is an association rule. Machine learning methods for association analysis include: Apriori algorithm, FP tree frequency set algorithm, and Upgrade Lift.

##### Apriori algorithm

The Apriori algorithm is based on the a priori principle and reflects the relationship between the subset and the superset: that is, all nonempty subsets of frequent itemsets must be frequent, and all supersets of infrequent sets must be infrequent. If item set I does not satisfy the minimum support thresholds, then I is not frequent. Frequent mode refers to the fact that the various items that appear in each shopping record actually reflect the nature of a combination. The combination of these items is unordered in the record, and this disordered combination is called “pattern.” Some of these modes have low frequency and some have high frequency. It is generally considered that the higher frequency is usually more instructive. This high frequency mode is called “frequent mode.” Therefore, the nature of the Apriori algorithm is mainly used to search for candidates when searching for frequent itemsets. Apriori algorithm can better avoid blind search and improve the efficiency of frequent item set search.

##### FP tree frequency set algorithm

The FP tree is constructed by reading in transactions one by one and mapping the transactions to a path in the FP tree. Since different transactions may have several identical items, their paths may partially overlap. The more the paths overlap each other, the better the compression effect obtained by using the FP tree structure; if the FP tree is small enough to be stored in the memory, the frequent itemsets can be extracted directly from the structure in the memory without having to repeatedly scan and store the data on the hard disk. The main idea of the FP tree frequency set algorithm is to compress the frequency set in the database into a frequent pattern tree after the first pass scan, while still retaining the associated information, and then separately mining the condition bases.

##### Upgrade lift

Regardless of the Apriori algorithm or the FP tree frequency set algorithm, in some cases, even if the two indicators of support and confidence are relatively high, the rules generated may still be useless. Lift gives a new indicator of the quality of the evaluation rules. Lift indicates the intensity of a given random occurrence of the predecessor and the back part, which provides an improved message to increase the probability of occurrence of the next piece of the given front piece.

#### 3.1.2 Cluster analysis

The classification algorithm must know the information of each category in advance, and all the data to be classified have corresponding categories. When the above conditions are not met, we need to try cluster analysis. Cluster analysis is to study how to classify similar things into one category. Clustering divides similar objects into different groups or more subsets by static classification, so that member objects in the same subset have similar properties. There are several clustering methods: partition‐based algorithm, hierarchical clustering algorithm, density‐based algorithm, and grid‐based algorithm.

##### Partition‐based algorithm

The K‐means method is the most commonly used and most basic clustering algorithm in cluster analysis. It is based on the prototype and partitioned distance technique. According to the given parameter K, the *N* objects are roughly divided into K classes, and then the unreasonable classification is modified according to some optimal principle. The advantages of the K‐means algorithm are that it is simple, fast, easy to understand, and has low time complexity. However, the K‐means are poorly processed for high‐dimensional data and do not recognize nonspherical clusters.

##### Hierarchical clustering algorithm

The hierarchical clustering algorithm hierarchically decomposes the data set. It is divided into agglomerative hierarchical clustering and top‐down divisive hierarchical clustering. Commonly used hierarchical clustering methods include BIRCH, CURE, ROCK, Chameleon, and other algorithms. This type of algorithm initially treats each point as a cluster. Clusters and clusters are combined according to closeness, and proximity can be defined differently according to the different meanings of “close.” The above combination process ends when further combinations result in undesired results under multiple causes.

##### Density‐based algorithm

In order to find clusters of arbitrary shape, the cluster can be regarded as a dense region separated by sparse regions in the data space, which is the core idea based on the density algorithm. Common methods include DBSCAN, OPTICS, and DENCLUE methods. DBSCAN is the most representative. It is a density‐based clustering based on high‐density connected regions, which connects core objects and their neighborhood‐difficult regions as clusters. Mainly used to deal with noise. The density of object O can be measured by the number of objects close to O. The core idea of the algorithm is to find all core points, boundary points, and noise points. DBSCAN does not need to input the number of clusters to be divided and can handle clusters of various shapes. However, the time complexity of the algorithm is high, so high‐dimensional data cannot be processed.

##### Grid‐based algorithm

Based on the partitioning and hierarchical clustering methods, the nonconvex shape clusters can not database. The algorithm that can effectively find the arbitrarily shaped clusters is based on the density algorithm, but the density‐based algorithm generally has a high time complexity. From 1996 to 2000, the research data mining scholars have proposed a large number of grid‐based clustering algorithms. The grid method can effectively reduce the computational complexity of the algorithm and is also sensitive to density parameters. The grid‐based clustering method uses a multiresolution grid data structure. The advantage of this method is that the processing speed is extremely fast and depends only on the number of elements in each dimension in the quantization space. Common methods include STING, CLIQUE, and WaveCluster. STING, based on grid multiresolution, space is divided into square units, corresponding to different resolutions; CLIQUE combined with the idea of the grid and density clustering, subspace clustering for high‐dimensional data; WaveCluster using wavelet analysis. The boundaries of the cluster become clearer.

### 3.2 Prediction

#### 3.2.1 Regression analysis

Traditional regression is a statistical analysis method that uses ordinary linear regression to determine the quantitative relationship between two or more variables. It is widely used. Its expression is *y* = *w*'*x *+ *e*, and *e* is a normal distribution with an error obeying a mean of 0. Regression analysis can be divided into a linear regression analysis and multiple linear regression analysis according to the number of independent variables. A linear regression analysis contains only one independent variable and one dependent variable, and a straight line can approximate the relationship between the two. If the regression analysis includes two or more independent variables, and the linear relationship between the dependent variable and the independent variable is called multiple linear regression analysis. In practice, a phenomenon is often associated with multiple factors. When performing regression analysis, you need two or more independent variables. This regression is called multiple regression. It is more effective and more realistic to predict or estimate the dependent variable by the optimal combination of multiple independent variables than to predict or estimate with only one independent variable. Therefore, multiple linear regression is more practical than one‐dimensional linear regression. Multiple linear regression analysis consists of three steps: the first step is using the collected data to establish a regression equation; second, performing a hypothesis test on the regression equation obtained by the analysis; third, when the regression equation has significant significance, it is necessary to perform the partial regression coefficient for each independent variable. Hypothesis testing, after eliminating the variables with no significant partial regression coefficients, re‐establish the multiple regression equations that do not contain the variables and repeat the above process. Its basic principle is to apply the least‐squares method to the regression of the linear regression model.

Most of the statistical models of traditional methods have specific requirements on the data, and the model itself has a mathematical form that can be clearly expressed. The pros and cons of the model are mostly judged according to the test obtained from the assumption of the distribution of the data. However, in the actual work process, it is difficult to make any assumptions about the distribution of data in the real world. At the same time, it is difficult to describe with a limited mathematical formula. The machine learning method has no assumptions about the data, and the results are also cross‐over. The method of verification judges that the prediction model based on the algorithm or program is quite effective and the result of cross‐validation is easily understood and accepted by the majority of practical workers. The machine learning methods for regression models are decision tree, adaptive boosting, bagging, random forests, support vector machines, nearest neighbor algorithm, and artificial neural network.

#### 3.2.2 Classification analysis

Classification is a supervised learning process. The goal is to “tag” the data to extract valuable data. The more accurate the categories are, the more valuable the results will be. Usually, the following methods are used: logistic regression, probit regression, classical discriminant analysis: there are not many classification levels of independent variables, and there are two levels of dependent variables; discriminant analysis: there are not many classification levels of independent variables, and there are more than two dependent variables level; machine learning method: there are many levels of independent variable classification. Building a classification model can help us better understand the data. However, there are limitations. The information of each category must be known in advance, and all the data to be classified have corresponding categories. When the dependent variable is a categorical variable and the independent variable contains multiple categorical variables or the categorical variable has a high level, the classical statistic is not applicable, and the machine learning method is more practical for processing complex data, and the accuracy is better.

## 4 PROSPECTS AND CHALLENGES OF MEDICAL DATA MINING

The use of new cutting‐edge disciplines to generate big data and analyze big data is a trend that has evolved between traditional medicine and precision medicine. The development of big data will help the global application of precision medicine and the emergence of new health management models.^27^ The potential for big data is still to be discovered. Although it is not easy to generate new findings and conclusions in massive amounts of data, as long as effective investments are made on the right systems, key breakthroughs in technology and workforce are available, and future big data analysis, visualization, and artificial intelligence can be foreseen. The convenience and change in medical care and life are worth looking forward to. The potential for big data is still to be discovered. However, medical big data mining still faces enormous challenges, mainly in the following: medical knowledge concept is complex, medical knowledge reasoning key technology has not broken through; medical information sources are wide, data modality is high, the latitude is high, the type is unbalanced, and structure is complicated. The hospital's electronic medical record system is poor in openness and scalability; the out‐of‐hospital process is poorly regulated. Although it is not easy to generate new findings and conclusions in massive data, we can foresee the future medical and life convenience of big data analysis as long as we make productive investments in appropriate systems and achieve key breakthroughs in technology and workforce.

## 5 CONCLUSIONS

This article first briefly introduces the database and data mining methods commonly used in the era of big data. With the advent of the information age, data mining is increasingly being used in clinical practice. With information technology, medical records and follow‐up data can be stored and extracted more efficiently. At the same time, look for potential relationships or patterns from medical data to gain useful knowledge of the diagnosis and treatment of patients. At the same time, it can also increase the predictive accuracy of the disease, find the disease at an early stage, and improve the cure rate. The database we introduced is only a small part, and there are many databases worthy of research attention, such as Catalogue of Somatic Mutations in Cancer (COSMIC), The Human Gene Mutation Database (HGMD), Oncomine, cBioPortal for Cancer Genomics (cBioPortal), Sequence Read Archive(SRA), WHO Mortality Database, Orphanet, Database of Genomic Variants (DGV), Online Mendelian Inheritance in Man (OMIM), etc. With the deepening of theoretical research and further practical exploration, medical data mining will play an influential role in the diagnosis and treatment of diseases, medical research and teaching, and hospital management.

## CONFLICT OF INTEREST

The authors declare no conflict of interest.
